# HOW’S YOUR MEMORY? CONVERGENCE OF AMBULATORY COGNITIVE PERFORMANCE AND RETROSPECTIVE PERFORMANCE REPORTS

**DOI:** 10.1093/geroni/igad104.1540

**Published:** 2023-12-21

**Authors:** Anqing Zheng, Tina Vo, Elizabeth Muñoz, Sally Wadsworth, Martin Sliwinski, Chandra Reynolds

**Affiliations:** University of Colorado Boulder, Boulder, Colorado, United States; University of California Riverside, Riverside, California, United States; The University of Texas at Austin, Austin, Texas, United States; University of Colorado Boulder, Boulder, Colorado, United States; Pennsylvania State University, State College, Pennsylvania, United States; University of Colorado Boulder, Boulder, Colorado, United States

## Abstract

Calibration accuracy, the agreement between individuals’ perceived performance and actual performance, plays a critical role in self-regulated learning, knowledge monitoring, and goal maintenance. However, it is unclear how calibration accuracy operates at the micro-level timescales, especially among individuals approaching midlife. We investigated the convergence between perceived cognitive performance and the trajectory of momentary assessments collected repeatedly over 14 days. We analyzed the data of 440 participants (Mage = 35.78, %Female = 57.7%) from the Colorado Adoption/Twin Study of Lifespan Behavioral Development and Cognitive Aging (CATSLife1), who completed spatial working memory tasks administered via smartphones up to three times a day. On average, participants’ working memory improved across the 14-day period (β=-.014, SE=.001, p<.001), with lower scores indicating more accurate placement and better performance. Moreover, we observed less day-to-day variability in working memory performance (β=-.010, SE=.001, p<.001). The trajectory of working memory performance was consistent with participants’ evaluations of cognitive performance after completing the study: those perceiving improvement in their performance tended to perform better overtime (r = .25, SE = .046) and showed less variability in momentary assessments (r = -.28, SE = .046). Participants’ self-reported performance was also positively correlated with their working memory intercept at the start of the study (r = .34, SE=.045). Exploratory analyses then examined the associations between momentary calibration accuracy and other personality characteristics (e.g., openness). Our findings suggest that understanding the dynamic interplay between calibration accuracy and cognitive performance could shed light on individual differences during periods of cognitive maintenance versus decline.

